# Detection of various fusion genes by one-step RT-PCR and the association with clinicopathological features in 242 cases of soft tissue tumor

**DOI:** 10.3389/fcell.2023.1214262

**Published:** 2023-08-09

**Authors:** Lingxie Song, Ying Zhang, Yuanyuan Wang, Qingxin Xia, Dandan Guo, Jiachen Cao, Xin Xin, Haoyue Cheng, Chunxia Liu, Xingyuan Jia, Feng Li

**Affiliations:** ^1^ Department of Pathology and Medical Research Center, Beijing Institute of Respiratory Medicine and Beijing Chao-Yang Hospital, Capital Medical University, Beijing, China; ^2^ Department of Pathology and Key Laboratory for Xinjiang Endemic and Ethnic Diseases, The First Affiliated Hospital, Shihezi University School of Medicine, Shihezi, China; ^3^ Affiliated Cancer Hospital of Zhengzhou University, Henan Cancer Hospital, Zhengzhou, Henan, China; ^4^ Department of Pathology, The Second Affiliated Hospital of Guangzhou Medical University, Guangzhou, China

**Keywords:** one-step reverse transcription-polymerase chain reaction, fluorescence *in situ* hybridization, RNA-sequencing, soft tissue tumor, fusion gene

## Abstract

**Introduction:** Over the past decades, an increasing number of chromosomal translocations have been found in different STSs, which not only has value for clinical diagnosis but also suggests the pathogenesis of STS. Fusion genes can be detected by FISH, RT-PCR, and next-generation sequencing. One-step RT-PCR is a convenient method to detect fusion genes with higher sensitivity and lower cost.

**Method:** In this study, 242 cases of soft tissue tumors were included, which were detected by one-step RT-PCR in multicenter with seven types of tumors: rhabdomyosarcoma (RMS), peripheral primitive neuroectodermal tumor (pPNET), synovial sarcoma (SS), myxoid liposarcomas (MLPS), alveolar soft part sarcoma (ASPS), dermatofibrosarcoma protuberans (DFSP), and soft tissue angiofibroma (AFST). 18 cases detected by one-step RT-PCR were further tested by FISH. One case with novel fusion gene detected by RNA-sequencing was further validated by one-step RT-PCR.

**Results:** The total positive rate of fusion genes was 60% (133/213) in the 242 samples detected by one-step RT-PCR, in which 29 samples could not be evaluated because of poor RNA quality. The positive rate of PAX3–FOXO1 was 88.6% (31/35) in alveolar rhabdomyosarcoma, EWSR1–FLI1 was 63% (17/27) in pPNET, SYT–SSX was 95.4% in SS (62/65), ASPSCR1–TFE3 was 100% in ASPS (10/10), FUS–DDIT3 was 80% in MLPS (4/5), and COL1A1–PDGFB was 66.7% in DFSP (8/12). For clinicopathological parameters, fusion gene status was correlated with age and location in 213 cases. The PAX3–FOXO1 fusion gene status was correlated with lymph node metastasis and distant metastasis in RMS. Furthermore, RMS patients with positive PAX3–FOXO1 fusion gene had a significantly shorter overall survival time than those patients with the negative fusion gene. Among them, the FISH result of 18 cases was concordant with one-step RT-PCR. As detected as the most common fusion types of AHRR–NCOA2 in one case of AFST were detected as negative by one-step RT-PCR. RNA-sequencing was used to determine the fusion genes, and a novel fusion gene PTCH1–PLAG1 was found. Moreover, the fusion gene was confirmed by one-step RT-PCR.

**Conclusion:** Our study indicates that one-step RT-PCR displays a reliable tool to detect fusion genes with the advantage of high accuracy and low cost. Moreover, it is a great tool to identify novel fusion genes. Overall, it provides useful information for molecular pathological diagnosis and improves the diagnosis rate of STSs.

## Highlights

- The fusion genes’ status of 242 cases of soft tissue tumor was detected by one-step RT-PCR using formalin-fixed paraffin-embedded samples.
- In rhabdomyosarcoma, the expression of PAX3–FOXO1 mRNA was correlated with lymph node metastasis and distant metastasis. The patients with a positive PAX3-FOXO1 fusion gene had a significantly short overall survival time.
- A novel fusion gene PTCH1–PLAG1 in AFST was discovered by RNA sequencing and was confirmed by one-step RT-PCR and FISH assay.

## Introduction

Sarcomas are a heterogeneous group of malignant neoplasms arising from mesenchymal cells. Sarcomas comprise 12%–15% of pediatric malignant tumors, although they are rare in adults (Stiller et al., 2013). Sarcomas have been classified into two large subgroups, bone sarcomas and soft tissue sarcomas (STSs). According to the WHO Classification of Tumors: Soft Tissue and Bone Tumors, 2020, the STS subgroup contains more than seventy subtypes, which comprises 70%–80% of all sarcomas and has the highest incidence among these relatively rare malignant tumors (Grunewald et al., 2020).

In recent decades, an increasing number of studies have found that chromosomal translocations and fusion genes occur in most STSs. Examples include the PAX3/PAX7–FOXO1 fusion gene in rhabdomyosarcoma (RMS) (Fredericks et al., 1995), the EWSR1 translocation in peripheral primitive neuroectodermal tumor (pPNET) and desmoplastic small round cell tumor (Chen et al., 2016), the TFE3–ASPL fusion gene in alveolar soft part sarcoma (ASPS) (Pradhan et al., 2015), and the SYT–SSX fusion gene in synovial sarcoma (SS) (Sun et al., 2008). Moreover, with the development of molecular technology, such as next-generation sequencing (NGS), reverse transcription-polymerase chain reaction (RT-PCR), and fluorescence *in situ* hybridization (FISH), an increasing number of newly discovered fusion genes have been reported in various soft tissues, such as the NUP160–SLC43A3 fusion gene in angiosarcoma (Shimozono et al., 2015) and the SRF–FOXO1/NCOA1 fusion gene in well-differentiated RMS (Karanian et al., 2020).

Therefore, the diagnosis of many STSs should be based not only on morphological characteristics and immunohistochemical results but also on molecular examination, such as FISH, RT-PCR, and NGS. For instance, the diagnosis of alveolar rhabdomyosarcoma (ARMS) is typically based on histopathological features, IHC results (most importantly desmin, MyoD1, and myogenin), and molecular results (RT-PCR for PAX3/PAX7–FOXO1 or FISH for PAX3/FOXO1 rearrangement).

Over the past 20 years, our research group has been engaged in research on fusion genes in soft tissue tumors, such as RMS, SS, pPNET, and ASPS (Zhou et al., 2017; Ju et al., 2018). We conducted one-step RT-PCR on hundreds of soft tissue sarcomas in various types. The purpose of this research is to retrospectively analyze STSs that were subjected to one-step RT-PCR.

## Materials and methods

### Case selection and study design

**FIGURE 1:**
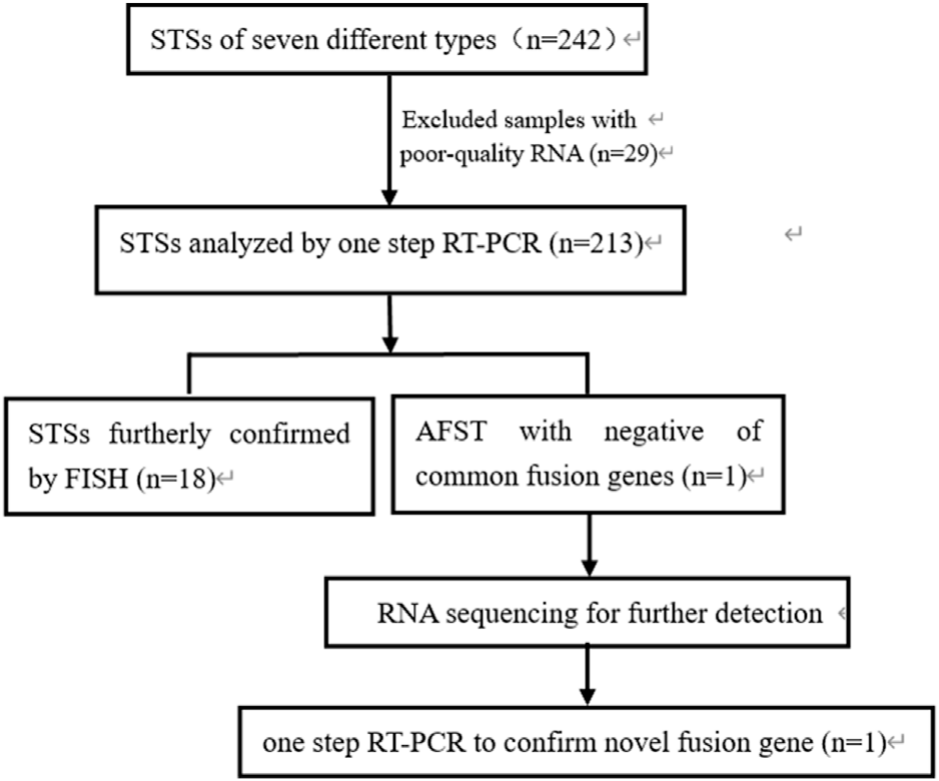
Flow chart showing the process of the study. STSs, soft tissue sarcomas; RNA, ribonucleic acid; RT-PCR, reverse transcription-polymerase chain reaction; FISH, fluorescence *in situ* hybridization; AFST, soft tissue angiofibroma.

This study included 242 cases of seven types of soft tissue tumors, and various fusion genes were detected by one-step RT-PCR. The formalin-fixed paraffin-embedded soft tissue tumor samples were collected from 1999 to 2021 at the First Affiliated Hospital, Shihezi University School of Medicine, Shihezi, XinJiang, China; Beijing Chaoyang Hospital, Capital Medical University, Beijing, China; and Affiliated Cancer Hospital of Zhengzhou University, Henan Cancer Hospital, Zhengzhou, China. These samples included 97 cases of RMS, 71 cases of SS, 42 cases of pPNET, 14 cases of ASPS, 12 cases of dermatofibrosarcoma protuberans (DFSP), five cases of myxoid liposarcomas (MLPS), and one case of angiofibroma of soft tissue (AFST). Meanwhile, 151 cases of 20 different types of tumor samples were used as negative controls, including lymphoma, malignant melanoma, and renal cell carcinoma. Clinical characterization was obtained from the case files, the electronic medical record system of the hospitals, and communication with patients. This study was approved by the Ethical Committee of the Beijing Chaoyang Hospital (approval number: 2022-5-26-3). Hematoxylin and eosin (H&E)-stained microsections were obtained for the initial diagnosis. Immunohistochemical (IHC) examination was conducted for significant majority of cases. The study process is schematically showed in Figure 1.

### RNA extraction and one-step RT-PCR analysis

Total RNA was isolated from 393 formalin-fixed paraffin-embedded (FFPE) tissues using TRIzol (cat. no. 15596018, Invitrogen). One-step RT-PCR analysis was conducted using a QIAGEN One-Step RT-PCR Kit (210212) for PAX3–FOXO1, EWSR1–FLI1, SYT–SSX, ASPSCR1–TFE3, FUS–DDIT3, COL1A1/PDGFB, AHRR–NCOA2, and PTCH1–PLAG1. EWSR1–FLI1 and ASPSCR1–TFE3 were detected using nested one-step RT-PCR. The reaction mixture consisted of the following: 5 × RT-PCR buffer 5.0 μL, 10.0 mM dNTP 1.0 μL, 10.0 mM forward primers 0.6 μL, 10.0 mM reverse primers 0.6 μL, enzyme mixed 0.5 μL, total RNA 2.0 μg, and added RNase-free water to a final volume of 25.0 μL. The PCR reaction was used for amplification using an ABI 9902 PCR thermocycler (Applied Biosystems). Amplified products were identified by electrophoresis in 2% agarose gel with ethidium bromide. Information of the primers used is shown in Table 1.

**TABLE 1:** Primer sequence used in the research.

Gene	Primer name	Sequences	Product length (bp)
PAX3–FOXO1	PAX3	5′- TAC​AGA​CAG​CTT​TGT​GCC​TC -3′	114
	FOXO1	5′- AAC​TTG​CTG​TGT​AGG​GAC​AG -3′	
EWSR1–FLI1	EWS exon 7	5′-TCC​TAC​AGC​CAA​GCT​CCA​AGT​C-3′	150–277
	FLI1 exon 9	5′-ACT​CCC​CGT​TGG​TCC​CCT​CC-3′	
SSX–SYT	SSX	5′-TTT​GTG​GGC​CAG​ATG​CTT​C-3’;	98
	SYT	5′-CCA​GCA​GAG​GCC​TTA​TGG​ATA-3’;	
COL1A1/PDGFB	COL1A1 (forward)		103–250
	Exon 11	5′-TCA​GGG​TGC​TCG​AGG​ATT​GC-3′	
	Exon 23	5′-AAG​CTG​GTC​GTC​CCG​GTG​AAG​C-3′	
	Exon 32	5′-TGAACGTGGT GTGA TCGTG G-3′	
	Exon 26	5′-AAG​GCT​GGA​GAG​CGA​GGT​GTT​C-3′	
	Exon 37	5′-TGC​TCC​TGG​AGC​CAA​AGG​TGC-3′	
	Exon 45	5′-TGG​CTT​CTC​TGG​CCT​CCA​GGG-3′	
	PDGFB (reverse)	5′-ATC​AAA​GGA​GCG​GAT​CGA​GTG​GTC-3′	
ASPL–TFE3	ASPL exon 7	5′-AAA​GAA​GTC​CAA​GTC​GGG​CCA-3′	300, 195
	TFE3 exon 6	5′-CGT​TTG​ATG​TTG​GGC​AGC​TCA-3′	
	ASPL (N) exon 7	5′-CGG​GCC​AGG​ATC​CCC​AGC​AG-3′	243, 138
	TFE3 (N) exon 6	5′-TGA​TGG​CTG​GTG​TGG​CCA​CG-3′	
FUS–DDIT3	FUS exon 5	5′-GCT​ATG​GAC​AGC​AGA​ACC​AGT-3′	111
	DDIT3 exon 2	5′-CTG​CTT​TCA​GGT​GTG​GTG​ATG-3′	
AHRR–NCOA2	AHRR exon 9	5′-ATT​GCG​GCA​CCC​GTT​CT-3′	143
	NCOA2 exon 16	5′-GGA​CAT​AGC​AAG​TCA​TCT​GGA​G-3′	
	AHRR exon 10	5′-GTC​TGT​GCG​AAT​CGG​AAC​TG-3′	94
	NCOA2 exon 14	5′-CAT​TCT​CCA​GAT​GGC​ATA​GTA​GGA-3′	
	NCOA2 exon 15	5′-GGA​CCT​CAG​TAT​AGC​CAA​CAA​C-3′	138
	AHRR exon 10	5′-CAG​TTC​CGA​TTC​GCA​CAG​AC-3′	
	NCOA2 exon 13	5′-AAG​GGA​TGA​TAG​GAA​ACC​AAG​G-3′	101
	AHRR exon 11	5′-GCC​AGC​GTC​AGT​CTG​TT-3′	
PTCH1–PLAG1	PTCH1	5′-TGA​TGT​GAA​ATC​CAA​GCC-3′	101
	PLAG1	5′-GAA​TCC​AAT​CCT​TCC​CAT​T-3′	
β-Actin	Actin forward	5′-GAGCGGGAA ATCGTCCGTGACATT-3′	234
	Actin reverse	5′-GAT​GGA​GTT​GAA​GGT​AGT​TTC​GTG-3′	

Note: N, nested PCR.

### Fluorescence *in situ* hybridization (FISH)

FISH analysis was performed on interphase nuclei of paraffin-embedded 3 μm sections using LSI dual-color break-apart probes specific for FOXO1 (FOXO1) at 13q14, SS18 (SYT) at 18q11.2, NCOA2 at 8q13, and PLAG1 at 8q12 (Anbiping, GuangZhou, China). One end of the probes was labeled with spectrum green (telomeric, 5′ to the breakpoint) and the other end with spectrum red (centromeric, 3′ to the breakpoint). FISH with the Anbiping probe was performed using the standard protocols supplied by the manufacturer. After deparaffinization in xylene and rehydration in a series of ethanol, the section was denatured in EDTA at 99°C (25 min), enzymatic digestion was carried out with pepsin solution at 37°C for 10 min, and finally, it was washed in 2 × SSC and passed through an alcohol series before incubation with the probes. Hybridization was performed overnight, according to the manufacturer’s protocols. After FISH, for each case and probe, a minimum of 100 non-overlapping nuclei, which were clearly identified and contained unequivocal signals, were counted. A break-part probe was considered to be split when the red and green signals were separated by two times the distance greater than the size of one hybridization signal. These break-apart rearrangements were interpreted as typical FISH patterns, while any other motifs were considered to be atypical. A specimen was considered positive if >15% of the nuclei showed a signal pattern consistent with the rearrangement.

### Sequencing analyses

RNA was extracted using an RNeasy FFPE Kit (73504, Qiagen, Hilden, Germany) from FFPE tumor tissue. RNA from the samples was used for RNA-seq. RNA libraries were then constructed using a TruSeq RNA Exome Kit (Illumina Inc., San Diego, United States). Finally, high-throughput sequencing was performed on the Illumina X10 platform (Illumina Inc., San Diego, CA, United States). Raw sequence reads were quality controlled using filter pipeline with multiple filtering steps as follows: 1) removing reads with adapters; 2) removing reads in which unknown bases were more than 5%; and 3) removing reads in which more than 15% of bases had low quality (sequencing quality no more than 19). After filtering, the remaining high-quality paired-end clean reads were retained for downstream bioinformatics analysis. High-quality reads were mapped to the reference genome hg19 via BowTie software (version 2.2.4) with default parameters. Gene fusion analysis was performed with two software tools, including STAR-Fusion (version 1.8.1, default parameters) and Arriba (version 1.2.0, default parameters). All the gene fusions were validated using Integrative Genomics Viewer (IGV) software.

### Statistical analysis

SPSS 19.0 software was used to analyze the data. χ2 or Fisher’s exact test and Kaplan–Meier analysis were used to calculate the data for the variables. *p* < 0.05 was considered statistically significant.

## Results

### Clinical and histopathological features of 242 cases of soft tissue tumor

In total, 242 cases of seven types of soft tissue tumors were included in our study. Patients with these tumors were 111 females and 131 males (F:M = :1.18), whose ages ranged from 6 months to 89 years (mean, 29 years). Tumor diameters of samples varied from 0.3 cm to 21 cm, in which tumor diameter greater than 5 cm was 118 (48.8%) cases. Notably, 147 of 242 (60.7%) cases occurred in the extremities and trunk region, 51 of 242 (21.1%) cases occurred in the head and neck, 14 of 242 (5.79%) cases occurred in the genitourinary tract, and 30 of 242 (12.4%) cases occurred in other sites including the thoracic cavity and parenchymal organs. Table 2 shows basic clinical characteristics information of 242 soft tissue tumor cases (detailed information is shown in Supplementary Table S1).

**TABLE 2:** Basic clinical characteristics of 242 cases of soft tissue tumors.

Characteristics	Cases	% of total
Gender		
Male	131	54.1
Female	111	45.9
Age (years)		
≤5	23	9.5
>5	219	90.5
Tumor diameter		
≤5 cm	118	48.8
>5 cm	124	51.2
Location		
Head and neck	51	21.1
Extremities and trunk	147	60.7
Genitourinary tract	14	5.79
Others	30	12.4
Tumor type		
RMS	97	40.1
pPNET	42	17.4
SS	71	29.3
ASPS	14	5.79
MLPS	5	2.07
DFSP	12	4.96
AFST	1	0.41

**FIGURE 2:**
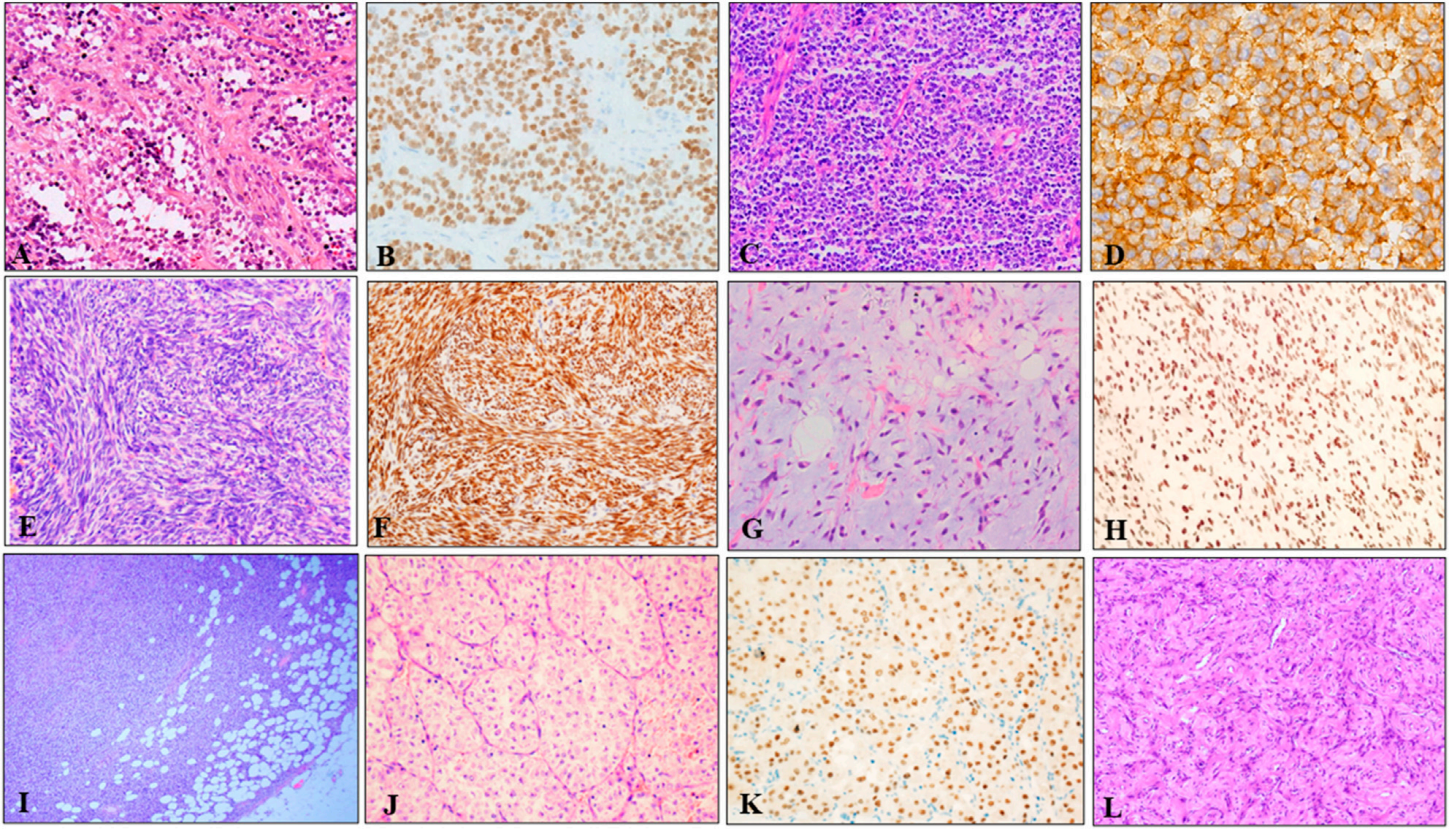
Representative HE and IHC of seven types of soft tissue tumors. **(A)** The tumor tissue was alveolar or nest-like, and the interstitium was fibrous vascular septum (HE × 100). **(B)** MyoD1 positive was evident in majority of tumor cells (×200). **(C)** pPNET was composed of uniform small blue round cells, showing vesicular nuclei with finely dispersed chromatin and scant cytoplasm (×100). **(D)** Immunohistochemistry showed diffuse, membranous CD99 positivity in pPNET (×400). **(E)** Microscopic images showed monophasic tumor entirely comprising spindle-cells arranged in bundles, eaves, and spirals (×200). **(F)** Immunohistochemistry demonstrated diffuse and strong nuclear staining for the transcriptional corepressor TLE1 (×200). **(G)** Microvacuolization in the myxoid stroma causing a lipoblast-like appearance was seen in some areas (×200). **(H)** Immunohistochemistry showed diffuse and nuclear expression of DDIT3 in MLSP (×200). **(I)** DFSP appeared as spindle cells diffusely infiltrating adipose tissue (×40). **(J)** ASPS appeared as typical organoid nests of eosinophilic tumor cells with abundant cytoplasm (×200). **(K)** Immunohistochemistry demonstrated strong nuclear staining for the TFE3 (×200). **(L)** AFST was composed of oval, short fusiform fibroblast-like cells, and abundant thin-walled branching blood vessels (×100).

These 242 soft tissue tumors diagnosed by morphology and immunohistochemistry in seven different types, included 97 cases of RMS, 71 of SS, 42 of pPNETs, 14 of ASPS, 12 of DFSP, five of MLPS, and one of AFST. On histopathological review of 39 ARMS among 97 cases of RMS, most ARMS displayed a characteristic alveolar structure that exhibited a loss of cellular cohesion in the center, and most tumor cells were primitive round with scant cytoplasm and hyperchromatic nuclei (Figure 2A). Immunohistochemically, tumor cells were positive for MyoD1 (Figure 2B) and desmin. Histologically, pPNET samples showed diffuse or leaflet arrangement with variable necrosis in some samples. The tumor cells were round or oval, with a clear nuclear membrane and fine nuclear chromatin, similar to salt and pepper (Figure 2C). Tumor cells expressed CD99 (Figure 2D) and Fli1. MFSS comprised short fascicles of spindle cells with eosinophilic cytoplasm and expressed TFE3 (Figures 2E, F). Figure 2G shows typical morphological features of MLPSs, with round, signet ring-like adipocytes, some of which are rich in mucus. Notably, DDIT3 IHC was nuclear positive for our MLPS cases (Figure 2H). DFSP displayed spindle tumor cells arranged in spiral and weave shapes infiltrating adipose tissue (Figure 2I). Morphologically, the ASPS had alveolar cells and contained eosinophilic red particles (Figure 2J). TFE3 IHC was nuclear positive for ASPS (Figure 2K). AFST was composed of short spindle, ovoid, and triangular cells, which were disposed within an extensively branching capillary network (Figure 2L).

### Detection of various fusion transcripts by one-step RT-PCR

A total of 242 soft tissue tumor samples were included in the study, 213 of which were detected by one-step RT-PCR, and other 29 samples could not be tested because of poor RNA quality. Table 3 summarizes the results of fusion transcripts detected by one-step RT-PCR in 213 soft tissue tumors in the study.

**TABLE 3:** Summary for the detection result of 213 cases of soft tissue tumors by one-step RT-PCR.

Tumor type	Number	Fusion gene	Positive	Negative	Positive rate (%)
RMS					
ARMS	35	PAX3–FOXO1	31	4	88.6% (31/35)
ERMS	43		0	43	0% (0/43)
PRMS	14		0	14	0% (0/14)
SRMS	1		0	1	0% (0/1)
pPNET	27	EWSR1–FLI1	17	10	63% (17/27)
SS	65	SYT–SSX	62	3	95.4% (62/65)
ASPS	10	ASPSCR1–TFE3	10	0	100% (10/10)
MLPS	5	FUS–DDIT3	4	1	80% (4/5)
DFSP	12	COL1A1–PDGFB	8	4	66.7% (8/12)
AFST	1	PTCH1–PLAG1	1	0	100% (1/1)
Total number	213		133	80	60% (133/213)

Note: RMS, rhabdomyosarcoma; ARMS, alveolar rhabdomyosarcoma; ERMS, embryonal rhabdomyosarcoma; PRMS, pleomorphic rhabdomyosarcoma; SRMS, sclerosing rhabdomyosarcoma; pPNET, peripheral primitive neuroectodermal tumor; SS, synovial sarcoma; ASPS, alveolar soft tissue sarcoma; MLPS, myxoid liposarcoma; DFSP, dermatofibrosarcoma protuberans; AFST, soft tissue angiofibroma.

**FIGURE 3:**
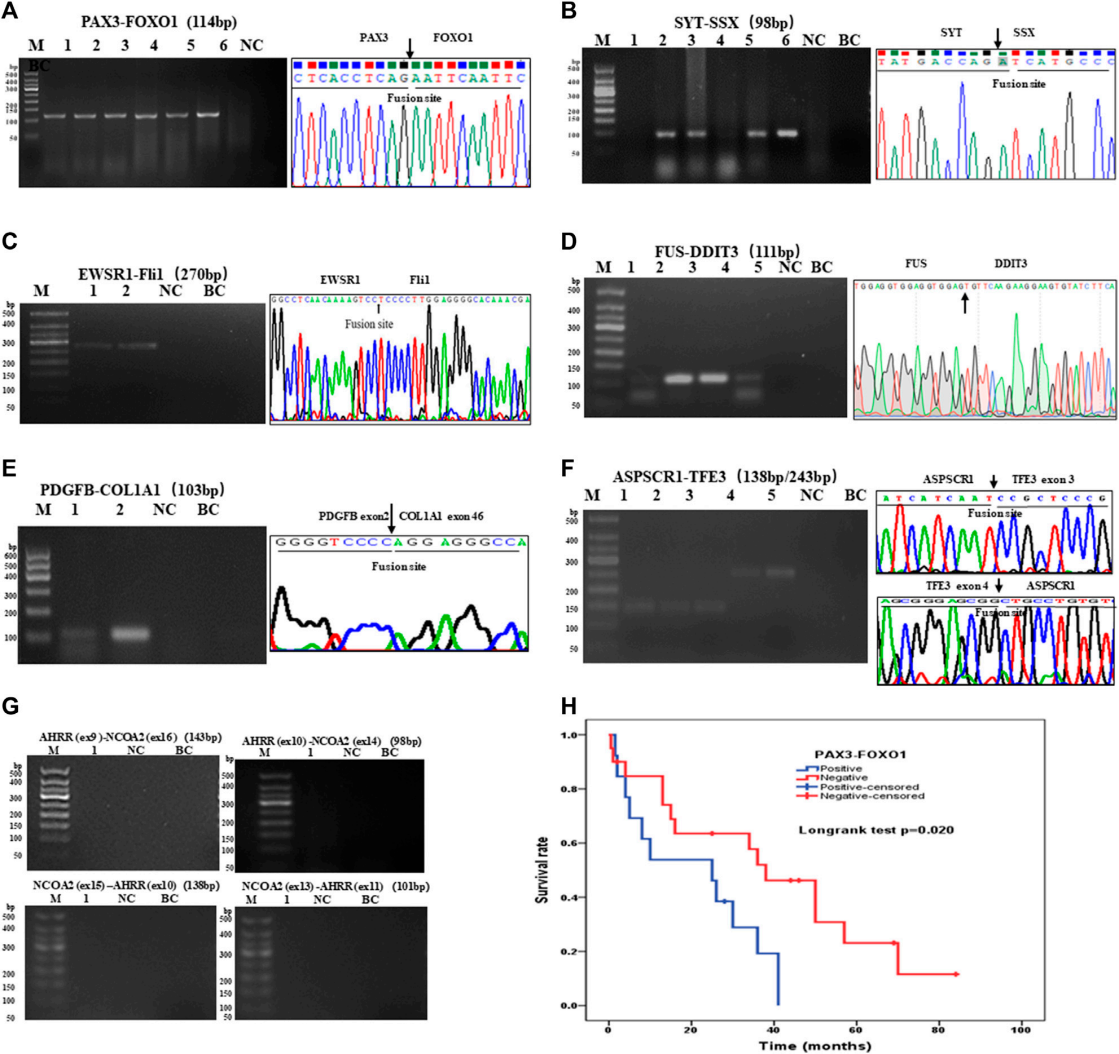
Agarose gel electrophoresis and sequencing image of one-step RT-PCR products of seven types of soft tissue tumors. **(A)** Agarose gel image of PAX3–FOXO1 amplification products (114-bp) of ARMS. Lanes 1–6, cases 1–6; lane 7, negative control; lane 8, blank control. **(B)** Agarose gel electrophoresis showing amplification products (98-bp) of SYT-SSX by one-step RT-PCR. Lanes 1–6, cases 1–6; lane 7, negative control; lane 8, blank control. **(C)** One-step RT-PCR detected the EWSR1–FLi1 fusion gene in pPNET. Lanes 1–2, cases 1–2; lane 3, negative control; lane 4, blank control. **(D)** FUS–DDIT3 fusion gene was detected in MLPS by one-step RT-PCR. Lanes 1–5, cases 1–5; **(E)** The electrophoresis images of one-step RT-PCR products of the COL1A1–PDGFB fusion gene in DFSP. Lanes 1–2, cases 1–2; lane 3, negative control; lane 4, blank control. **(F)** Electrophoresis and sequencing image of one-step RT-PCR products (138-bp, 243-bp) of ASPSCR1–TFE3 (exon 3/exon 4). Lanes 1–5, cases 1–5; lane 6 negative control; lane 7, blank control. **(G)** One-step RT-PCR performed on AFST detecting no AHRR–NCOA2 fusion gene. Lane 1, cases 1; lane 2, negative control; lane 3, blank control. **(H)** Kaplan–Meier analysis of correlations between PAX3–FOXO1 fusion gene and OS (overall survival time) of RMS patients.

There were 97 cases of RMS including 39 of ARMS, 43 of ERMS, 14 of PRMS, and one of SRMS, in which 93 samples were tested for PAX3–FOXO1 fusion transcripts by one-step RT-PCR, and other four samples could not be tested because of poor RNA quality. Among 93 cases of RMS, 31 cases of ARMS were positive for PAX3–FOXO1 transcript with a length of 114-bp product by one-step RT-PCR (Figure 3A). Among 71 cases of SS, six cases failed to be tested for SYT–SSX fusion transcript because of poor RNA quality, and 62 cases were positive with a 98-bp product. Figure 3B shows that cases of No. 2, 3, 5 and 6 were identified as SYT–SSX fusion transcript positive, and cases of No.1 and 4 were identified as negative. Among 42 cases of pPNET, 15 cases were with poor RNA quality, and 17 cases were positive for EWSR1–FLI1 fusion transcript by one-step RT-PCR (Figure 3C). All five cases of MLPS were detected for FUS–DDIT3 fusion transcript by one-step RT-PCR, in which four cases were positive with a 111-bp amplification product (Figure 3D). In total, 12 cases of DFSP were detected for COL1A1–PDGFB fusion transcript, in which eight cases were determined as positive. Figure 3E shows the transcript of COL1A1 (exon 46)–PDGFB (exon 2) fusion gene. Among 14 cases of ASPS, 10 cases were determined as positive for ASPSCR1–TFE3 (exon 3/exon 4) transcripts by one-step RT-PCR (Figure 3F), while four cases failed to be tested because of poor RNA quality. In one case of AFST, the common four transcripts of AHRR–NCOA2 fusion genes were determined as negative by one-step RT-PCR (Figure 3G).

Furthermore, we analyzed the association between fusion gene status and the clinicopathological parameters in 213 soft tissue tumors. Table 4 shows that the fusion gene status in total was correlated with age (χ2 = 7.114, *p* = 0.008) and location (χ2 = 14.712, *p* = 0.002) but was not related to gender and tumor diameter (Table 4). Table 5 reveals the correlation between PAX3–FOXO1 fusion gene status and clinicopathological parameters in RMS patients. After analysis, we found that the PAX3–FOXO1 fusion gene status was correlated with histologic type (χ2 = 85.363, *p* = 0), lymph node metastasis (χ2 = 8.942, *p* = 0.003), and distant metastasis (χ2 = 6.082, *p* = 0.014). Furthermore, from Kaplan–Meier analysis, we found that RMS patients with positive PAX3–FOXO1 fusion gene had a significantly shorter overall survival time than those patients with the negative fusion gene (Figure 3H). In addition, the correlation between SYT–SSX fusion gene status and clinicopathological parameters was analyzed. We found that the SYT–SSX fusion gene status was not correlated with gender, tumor diameter, location, and histologic type (Table 6).

**TABLE 4:** Association between the fusion gene status and clinicopathological parameters in 213 cases of soft tissue tumors.

Variables	Cases	Fusion gene			
		Negatives	Positives	*X* ^2^	*p*
Gender					
Male	114 (53.5%)	44 (20.7%)	70 (32.9%)	0.113	0.737
Female	99 (46.5%)	36 (16.9%)	63 (29.6%)		
Age (years)					
≤5	22 (10.3%)	14 (6.57%)	8 (3.76%)	7.114	0.008*
>5	191 (89.7%)	66 (31%)	125 (58.7%)		
Tumor diameter					
≤5 cm	101 (47.4%)	41 (19.2%)	60 (28.2%)	0.755	0.385
>5 cm	112 (52.6%)	39 (18.3%)	73 (34.3%)		
Location					
Head and neck	43 (20.2%)	24 (11.3%)	19 (8.92%)	14.712	0.002*
Extremities and trunk	129 (60.6%)	37 (17.4%)	92 (43.2%)		
Genitourinary tract	14 (6.57%)	9 (4.23%)	5 (2.35%)		
Others	27 (12.7%)	10 (4.69%)	17 (7.98%)		

**TABLE 5:** Association between the PAX3–FOXO1 fusion gene status and clinicopathological parameters in 93 cases of RMS.

Variables total	Cases	PAX3–FOXO1			
		Negative	Positives	*X* ^*2*^	*p*
Gender					
Male	53 (57%)	36 (38.7%)	17 (18.3%)	0.088	0.776
Female	40 (43%)	26 (28%)	14 (15%)		
Age (years)					
≤5	21 (22.6%)	14 (15.1%)	7 (7.5%)	0	1
>5	72 (77.4%)	48 (51.6%)	24 (25.8%)		
Tumor diameter					
≤5 cm	55 (59.1%)	34 (36.6%)	21 (22.6%)	1.424	0.233
>5 cm	38 (40.9%)	28 (30.1%)	10 (10.8%)		
Location					
Head and neck	36 (38.7%)	24 (25.8%)	12 (12.9%)	1.197	0.721
Extremities and trunk	33 (35.5%)	20 (21.5%)	13 (14%)		
Genitourinary tract	12 (12.9%)	9 (9.7%)	3 (3.2%)		
Others	12 (12.9%)	9 (9.7%)	3 (3.2%)		
Histologic type					
ARMS	35 (37.6%)	4 (4.3%)	31 (33.3%)	85.363	0*
ERMS	43 (46.2%)	43 (46.2%)	0 (0%)		
PRMS	14 (15.1%)	14 (15.1%)	0 (0%)		
SRMS	1 (1.08%)	1 (1.08%)	0 (0%)		
Histological grading					
I	35 (37.6%)	28 (30.1%)	7 (7.5%)	4.490	0.106
II	46 (49.5%)	27 (29%)	19 (20.4%)		
III	12 (12.9%)	7 (7.5%)	5 (5.4%)		
TNM stage					
I–II	59 (63.4%)	40 (43%)	19 (20.4%)	0.093	0.821
Ⅲ-Ⅳ	34 (36.6%)	22 (23.7%)	12 (12.9%)		
Lymph node metastasis					
No	78 (83.9%)	57 (61.3%)	21 (22.6%)	8.942	0.003*
Yes	15 (16.1%)	5 (5.38%)	10 (10.75)		
Distant metastasis					
No	76 (81.7%)	55 (59.1%)	21 (22.6%)	6.082	0.014*
Yes	17 (18.3%)	7 (7.52%)	10 (10.75)		

**TABLE 6:** Association between the SYT–SSX fusion gene status and clinicopathological parameters in 65 cases of SS.

Variables	Cases	SYT–SSX		*X* ^*2*^	*p*
		Negatives	Positives		
Gender					
Male	36 (55.4%)	1 (1.54%)	35 (53.8%)		0.582
Female	29 (44.6%)	2 (30.1%)	27 (41.5%)		
Age (years)					
≤35	36 (55.4%)	2 (3.08%)	34 (52.3%)		1
>35	29 (44.6%)	1 (1.54%)	28 (43.1%)		
Tumor diameter					
≤5 cm	31 (47.7%)	3 (4.62%)	28 (43.1%)		0.103
>5 cm	34 (52.3%)	0 (0%)	34 (52.3%)		
Location					
Head and neck	6 (9.23%)	0 (0%)	6 (9.23%)	1.995	1
Extremities and trunk	54 (83.1%)	3 (4.62%)	51 (78.5%)		
Genitourinary tract	1 (1.54%)	0 (0%)	1 (1.54%)		
Others	4 (6.15%)	0 (0%)	4 (6.15%)		
Histologic type					
BSS	36 (55.4%)	1 (1.54%)	35 (53.8%)	2.582	0.296
MFSS	23 (35.4%)	1 (1.54%)	22 (33.8%)		
PDFF	6 (9.23%)	1 (1.54%)	5 (7.69%)		

### FISH validation for fusion transcripts detected by one-step RT-PCR

**FIGURE 4:**
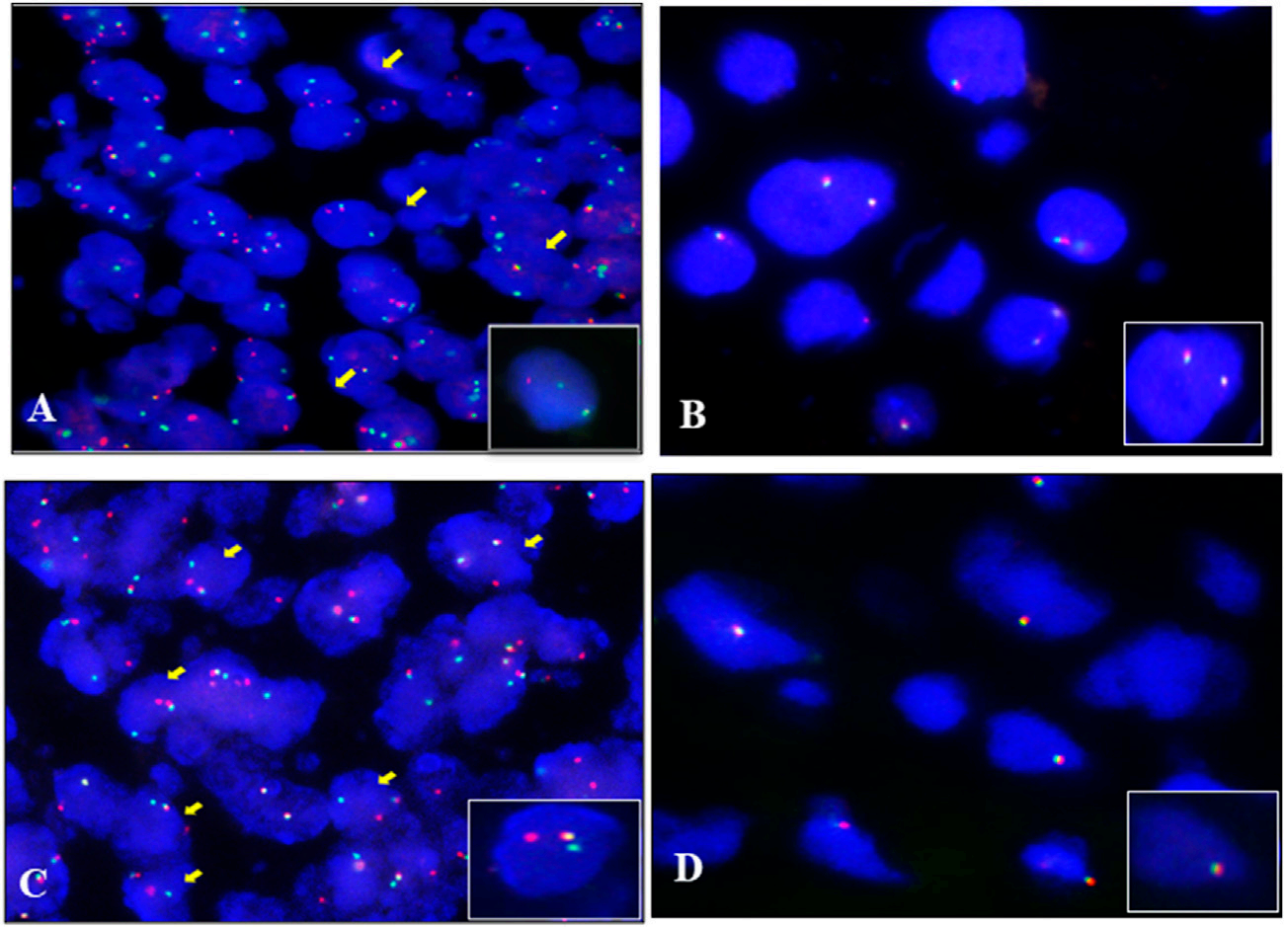
Fluorescence *in situ* hybridization (FISH) examination of the seven types of soft tissue tumors. **(A)** ARMS showing typical FOXO1 rearrangement with one fusion, one red, and one green signal (1F1R1G). **(B)** Negative signal of FOXO1 rearrangement (2F) examined by the FISH method in ERMS. **(C)** Typical image of SS18 rearrangement (1F1R1G) of FISH in SS. **(D)** Negative signal of NCOA2 rearrangement (2F) examined by the FISH method in AFST.

To validate the result of fusion transcripts detected by one-step RT-PCR, FISH was used to test 18 cases of soft tissue tumors (included 14 cases of RMS, three cases of SS, and one case of AFST). FISH results showed that FOXO1 rearrangement was positive in 10 ARMS (Figure 4A). FOXO1 rearrangement was negative in four ERMS (Figure 4B). Figure 4C shows that 1F1R1G indicating one fusion, one red signal, and one green signal, which referred to a typical positive signal of the SYT rearrangement in all three samples of SS. To confirm the case with the negative result of AHRR–NCOA2 of AFST detected by one-step RT-PCR, we used NCOA2 separation FISH probe to detect the rearrangement and found NCOA2 rearrangement to be negative (Figure 4D). These results were consistent with that of one-step RT-PCR (Supplementary Table S1).

### Identification of a novel fusion gene in AFST by one-step RT-PCR

**FIGURE 5:**
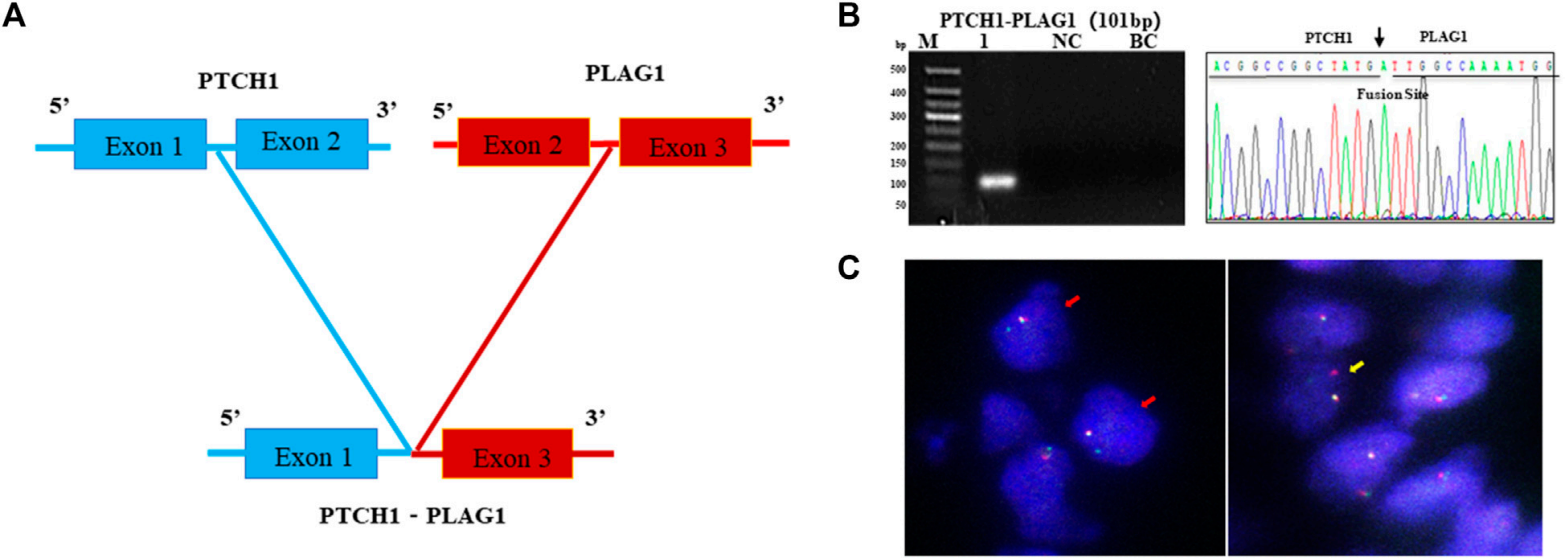
A novel PTCH1-PLAG1 fusion gene identified by one-step RT-PCR in AFST. **(A)** The PTCH1–PLAG1 fusion gene between PTCH1 exon 1 and PLAG1 exon 3 examined by RNA sequencing in AFST. **(B)** Agarose gel electrophoresis showing one-step RT-PCR amplification products of PTCH1–PLAG1. Lane 1, positive for PTCH1-PLAG1 in 101-bp; lane 2, negative control; lane 3, blank control. **(C)** PLAG1 rearrangement detected with typical 1F1R1G (yellow arrow) and atypical 1F1G (red arrow) on FISH.

As the most common AHRR–NCOA2 fusion transcript types of AFST were determined as negative by one-step RT-PCR detection, and NCOA2 rearrangement in AFST was determined as negative by FISH, the AFST sample was further assayed by RNA-sequencing. A novel PTCH1–PLAG1 fusion gene between PTCH1 exon 1 and PLAG1 exon 3 was discovered (Figure 5A). To verify the result of RNA-sequencing, we further examined PTCH1–PLAG1 fusion gene by one-step RT-PCR. The result of agarose gel electrophoresis showed a specific 101-bp amplification product, and the product was confirmed by DNA sequencing (Figure 5B). PLAG1 separation FISH probe was used to detect PLAG1 arrangement and FISH showed a positive result with typical (1F1R1G) signals for the arrangement (Figure 5C). Thus, the novel fusion gene of PTCH1–PLAG1 was determined.

## Discussion

Over the past three decades, a number of studies have shown that chromosome translocation and their fusion genes are closely related to the occurrence and development of various cancers (Mertens et al., 2015). The detection of fusion genes is of great significance for the diagnosis of these tumors. Conventional techniques to detect gene fusions are CISH, RT-PCR, and FISH. With the development of molecular technology, many new molecular diagnostic assays such as next-generation sequencing have been developed for detection of gene fusions in sarcoma (Lanic et al., 2022). In comparison with conventional RT-PCR analysis, one-step RT-PCR runs reverse transcription and PCR amplification in the same tube (Nikiforova et al., 2005). It is simple, time-saving, efficient, and reduces the risk of contamination and is particularly suitable for paraffin embedded tissues and to meet the needs of routine clinical pathological diagnosis work. So far, studies on the detection of soft tissue tumors by one-step RT-PCR was limited, and most of them had a small sample size or detected only one or few types of tumors (Yang et al., 2012; Norlelawati et al., 2016). Motegi et al. detected fusion transcripts of COL1A1–PDGFB in five DFSP samples using one-step RT-PCR (Yokoyama et al., 2012). Wang et al. applied one-step RT-PCR to detect PAX3–FOXO1 fusion gene in 13 ARMS samples (Yang et al., 2012). Our research reports a relatively large group of STSs (242) tested by one-step RT-PCR. We retrospectively summarized the morphological characteristics, clinical parameter characteristics, and molecular features of seven different types of 242 cases of STSs. The overall incidence in males was slightly higher than that in females (1:1.18). The mean age at diagnosis was 29 years, and the extremities and trunk region were the most common tumor sites, with a proportion of 60.7%, which was in line with the literature (Stiller et al., 2013). Because some of our cases were consultation cases and the samples were returned, some information about histological grading and prognosis were missed.

A total of 213 cases of STSs samples were detected by one-step RT-PCR in our study; 29 cases were not applicable because of poor RNA quality. The total positive rate of fusion transcripts was 60% (133/213) in 213 cases of STSs samples. We performed one-step RT-PCR to detect PAX3–FOXO1 fusion gene in 93 cases of RMS samples, with a positive rate of 88.6% in 35 cases of ARMS. The positive rate of EWSR1–FLI1 was 63% (17/27) in pPNET. A total of 65 cases of SS were included to detect SYT–SSX with a positive rate of 95.4% (62/65). In addition, the positive rate of ASPSCR1–TFE3 in ASPS, FUS–DDIT3 in MLPS, and COL1A1–PDGFB in DFSP were 100% (10/10), 80% (4/5), and 66.7% (8/12), respectively. We analyzed the association between fusion gene status and the clinicopathological parameters in 213 cases of soft tissue tumors. PAX3–FOXO1 in ARMS correlated with lymph node metastasis and distant metastasis and related to patients’ overall survival time. Among the 213 cases of STSs samples, 18 cases were further examined by FISH. The result of one-step RT-PCR for fusion transcripts was consistent with that of FISH.

Routinely, the preliminary diagnosis of RMS is based on typical morphological characteristics with representative cytoplasm desmin positivity, along with nuclear MyoD1 and myogenin positivity (Rekhi et al., 2018). In our study, we preliminarily diagnosed RMS by histomorphology and IHC. We performed further molecular testing by one-step RT-PCR in 93 cases of RMS, of which 31 cases were positive for PAX3–FOXO1, with a positive rate of 88.6% in 35 cases of ARMS. Frederic previously detected the fusion gene PAX3–FOXO1 in 78 cases of ARMS, and 43 cases were positive for PAX3–FOXO1, with a positive rate of 55%, which had a lower positive rate in their study (Sorensen et al., 2002). We detected FOXO1 rearrangement by FISH in 14 cases of RMS samples, a typical positive signal (1F1R1G) was observed in 10 cases of ARMS (Fu et al., 2017). The PAX3–FOXO1 fusion gene status was correlated with the TNM stage, lymph node metastasis, and distant metastasis. Moreover, PAX3–FOXO1 positive RMS patients had a significantly shorter survival period than PAX3–FOXO1 negative RMS patients.

42 cases of pPNET samples included in our study were diagnosed by histology and immunophenotype features. Some cases underwent immunostaining for vimentin, CD99, and Fli1, while a few cases for NKX2.2 (Russell-Goldman et al., 2018). In pPNET, 85%–90% of cases were found to have the EWSR1–FLI1 fusion gene as a chromosomal translocation t (11; 22) (q24; q12) (Khoury, 2005), while other variant translocations lead to specific chimeric transcripts such as EWS–ERG, EWS–ETV1, EWS–E1AF, EWS–FEV, EWS–PATZ1, EWS–SP3, FUS–ERG, and FUS–FEV (Zucman et al., 1993; Machado et al., 2009). In our research, we carried out one-step RT-PCR in 27 cases of pPNET, and 17 cases were positive for EWSR1–FLI1.

Cytogenetically, SS is characterized by the translocation t (X; 18) (p11.2; q11.2) to form the fusion gene SYT–SSX (Ladanyi, 2001). A total of 75 cases of SS samples were preliminarily diagnosed by morphology and IHC features. Then, 65 cases of SS samples were tested by one-step RT-PCR, of which 62 cases were positive for SYT–SSX1/SSX2 (95.5%), and three cases of SS samples were tested by FISH, of which three cases were positive for SYT rearrangement (100%). The positive ratio of one-step RT-PCR and FISH was similar to those reported in the literature (Sun et al., 2008; Ten Heuvel et al., 2008). The clinicopathological parameters showed that the SYT–SSX fusion gene status was not related to gender, tumor diameter, location, and histologic type, which was consistent with the previous research study (Sun et al., 2009).

The FUS–DDIT3 fusion gene in MLPS was first reported in 1993 (Crozat et al., 1993). We found that three cases of MLPS samples were FUS-DDIT3 positive among five cases with typical histological features and immunohistochemical expression of S100 protein. In addition, we performed immunostaining of DDIT3 in two MLPS cases, since it was reported to be a useful marker for MLPS (Baranov et al., 2021). ASPS is a rare malignant soft tissue tumor that occurs in adolescents aged 15–35 years (Wang et al., 2014). The ASPL–TFE3 fusion gene as a chromosome translocation of t (X; 17) was first detected in ASPS in 2001 (Ladanyi et al., 2001). In our previous study, we have tested ASPL–TFE3 fusion gene in nine cases of ASPS samples (Ju et al., 2018). In this research, 10 cases of ASPS were successfully detected by one-step RT-PCR, and they were all positive for the ASPL–TFE3 fusion gene.

DFSP is a soft tissue tumor that was first named by Darier and Ferrand in 1924, and it carries a specific chromosomal translocation to form the COL1A1–PDGFB fusion gene (O'Brien et al., 1998). It was reported that the PDGFB exon 2 could be fused with variable COL1A1 exons from exons 6 to 43 (Maire et al., 2002; Llombart et al., 2011; Yokoyama et al., 2012; Nakamura et al., 2015). We have tested COL1A1–PDGFB fusion gene in 12 cases of DFSP samples in our previous study (Li et al., 2004). Eight of 12 cases of DFSP samples were positive for COL1A1–PDGFB transcripts detected by one-step RT-PCR.

AFST is a newly recognized benign fibrovascular tumor with unique clinical pathological and genetic characteristics which was first reported by Fletcher on 2012. The AHRR–NCOA2/NCOA2–AHRR fusion gene was the most common fusion gene as chromosome translocation of t (5; 8) in AFST. Interestingly, the most common fusion transcripts of AFST were detected as negative by one-step RT-PCR and FISH; however, a novel PTCH1–PLAG1 fusion gene was found by RNA-sequencing and was successfully confirmed by one-step RT-PCR and FISH, respectively. The human PTCH1 gene locates on chromosome 9q22.3 (Skoda et al., 2018). PTCH1 is called a tumor suppressor gene, and it plays a role in cell growth, cell specialization, and determining the shape of many different parts of the developing body (Huang et al., 2022). PLAG1 locates on 8q11, and it is predicted to have the activity of transcription factor. Hosoi et al. identified that COL3A1 and RAB2A can be novel translocation partner genes for PLAG1 in lipoblastoma (Nitta et al., 2019). So far, there have been no reports on the PTCH1–PLAG1 fusion gene. The novel PTCH1–PLAG1 fusion gene was detected by RNA sequencing at the first and then was confirmed by one-step RT-PCR and FISH in our study.

In summary, we retrospectively analyzed the morphological characteristics, molecular traits, and clinical parameter characteristics of seven types of soft tissue sarcoma in 242 cases of soft tissue sarcoma in our pathology database. Among the 242 cases, of which 213 were successfully detected by one-step RT-PCR. It indicated one-step RT-PCR for detecting fusion genes in paraffin tissue is of great significance for clinical pathological diagnosis. We also analyzed the association between fusion gene status and clinicopathological features. Eighteen cases were tested by FISH furtherly, and the result was consistent with that of one-step RT-PCR. Interestingly, a novel PTCH1–PLAG1 fusion gene has been discovered by RNA sequencing. We confirmed it by using one-step RT-PCR and FISH. These findings indicate that one-step RT-PCR can be used not only to detect known fusion genes for clinical pathological diagnosis but also to validate new fusion genes discovered by sequencing.

There are some limitations in the study. First, we could not carry out FISH in more cases of 242 samples to validate the result of fusion transcripts detected by one-step RT-PCR, partly, because of samples not available in some consultation cases. Second, there are only few samples detected and analyzed by NGS in the study. Thus, further large-scale and deep research is needed to eliminate these drawbacks.

## Data Availability

The datasets presented in this study can be found in online repositories. The names of the repository/repositories and accession number(s) can be found below: National Genomics Data Centre under HRA005141.

## Abbreviations

AFST, soft tissue angiofibroma; ASPS, alveolar soft part sarcoma; DFPS, dermatofibrosarcoma protuberans; FISH, fluorescence *in situ* hybridization; MLPS, myxoid liposarcoma; NGS, next-generation sequencing; pPNET, peripheral primitive neuroectodermal tumor; RMS, rhabdomyosarcoma; SS, synovial sarcoma; STSs, soft tissue sarcomas.
