# Supplementary material for: Detection of various fusion genes by one-step RT-PCR and the association with clinicopathological features in 242 cases of soft tissue tumor
Source: Front Cell Dev Biol. 2023 Aug 9;11:1214262. doi: 10.3389/fcell.2023.1214262 (PMC10446835; doi:10.3389/fcell.2023.1214262)
Supplement: Supplementary file 1 [file Table1.docx]

**Supplementary Table 1. Clinicopathological characteristics in 242 cases of soft tissue tumors**

| **Patient ID** | **Tumor type** | **Gender** | **Age (y)** | **Size**  **(cm）** | **Location** | **TNM stage** | **Histological grading** | **Lymph node metastasis** | **Distant metastasis** | **Fusion gene** | |
| --- | --- | --- | --- | --- | --- | --- | --- | --- | --- | --- | --- |
|  |  |  |  |  |  |  |  |  |  | **One-step**  **RT-PCR** | **FISH** |
| 1 | ERMS | F | 3 | 21 | throat | I | I | No | No | Negative | NA |
| 2 | ERMS | M | 19 | 2 | throat | Ⅳ | II | No | Yes | Negative | NA |
| 3 | ARMS | M | 17 | 2 | upper left abdomen | Ⅳ | Ⅲ | No | Yes | Positive | NA |
| 4 | ERMS | M | 40 | 1 | left nasal cavity | I | I | No | No | Negative | NA |
| 5 | ERMS | F | 54 | 3 | lymph node | I | I | No | No | Negative | NA |
| 6 | ARMS | F | 56 | 1 | left orbital | Ⅲ | I | Yes | No | Positive | NA |
| 7 | ERMS | F | 31 | 3 | nasopharynx | II | II | No | No | Negative | NA |
| 8 | ARMS | M | 18 | 5 | left forearm | I | II | No | No | Positive | NA |
| 9 | ERMS | M | 2 | 3 | right eye frame | I | II | No | No | Negative | NA |
| 10 | ERMS | M | 4 | 5 | bladder | II | I | No | No | Negative | NA |
| 11 | ERMS | M | 4 | 3.5 | left epididymis | I | I | No | No | Negative | NA |
| 12 | ARMS | F | 10 | 2.2 | right cervical lymph nodes | Ⅳ | I | Yes | Yes | Positive | NA |
| 13 | ARMS | F | 14 | 4 | left parotid gland | II | I | No | No | Positive | NA |
| 14 | ERMS | F | 42 | 19 | retroperitoneum | Ⅲ | II | No | No | Negative | NA |
| 15 | ERMS | F | 2 | 1.8 | right infraorbital area paranasal | Ⅳ | I | No | Yes | Negative | NA |
| 16 | ERMS | F | 54 | 2.1 | maxillary sinus | I | II | No | No | Negative | NA |
| 17 | ERMS | M | 40 | 3 | left nasal cavity | I | I | No | No | Negative | NA |
| 18 | ERMS | F | 13 | 3 | left parotid gland | I | I | No | No | Negative | NA |
| 19 | ERMS | F | 13 | 9 | left cheek tumor | Ⅲ | II | No | No | Negative | NA |
| 20 | ARMS | F | 13 | 2 | limbs torso | Ⅳ | I | Yes | Yes | Positive | NA |
| 21 | ERMS | M | 0.67 | 0.5 | bladder | Ⅳ | I | Yes | No | Negative | NA |
| 22 | ERMS | F | 39 | 6 | retroperitoneum | I | I | No | No | Negative | NA |
| 23 | ARMS | F | 9 | 10.3 | groin | I | I | No | No | Negative | NA |
| 24 | ERMS | M | 5 | 10 | bladder | I | I | No | No | Negative | NA |
| 25 | ARMS | M | 15 | 6.5 | ulnar side of right forearm | I | I | No | No | Positive | NA |
| 26 | ARMS | M | 17 | 9 | right upper arm | I | II | No | No | Positive | NA |
| 27 | ARMS | F | 3 | 1 | left neck | I | II | No | No | Positive | NA |
| 28 | ERMS | M | 51 | 0.2 | right inguinal lymph node | I | II | No | No | Negative | NA |
| 29 | PRMS | F | 64 | 4 | right upper arm | I | I | No | No | Negative | NA |
| 30 | PRMS | M | 48 | 15 | left hip | Ⅳ | I | No | No | Negative | NA |
| 31 | ERMS | M | 2 | 10 | buttocks | Ⅳ | I | No | Yes | Negative | NA |
| 32 | ERMS | M | 3 | 2 | left nasal cavity | I | II | No | No | Negative | NA |
| 33 | ARMS | F | 18 | 6.7 | right forehead | I | I | No | No | Negative | NA |
| 34 | ERMS | F | 18 | 12 | right lower limb | Ⅲ | I | No | No | Negative | NA |
| 35 | ERMS | M | 4 | 5.5 | abdominal wall | Ⅳ | II | No | Yes | Negative | NA |
| 36 | ARMS | F | 3 | 2.8 | upper lip | II | I | Yes | Yes | Positive | NA |
| 37 | PRMS | M | 55 | 7.6 | left thigh | II | I | No | No | Negative | NA |
| 38 | PRMS | M | 69 | 11 | right armpit | Ⅲ | II | No | No | Negative | NA |
| 39 | ERMS | M | 20 | 5.5 | right kidney | I | I | No | No | Negative | NA |
| 40 | ERMS | F | 8 | 1.2 | right pharynx | I | II | No | No | Negative | NA |
| 41 | ERMS | M | 16 | 6.3 | left scrotum | Ⅳ | II | Yes | No | Negative | NA |
| 42 | ERMS | M | 29 | 2.5 | buccal gums | Ⅳ | I | No | Yes | Negative | NA |
| 43 | ARMS | F | 5 | 13 | right thigh | Ⅲ | I | Yes | Yes | Positive | NA |
| 44 | ARMS | F | 46 | 2.3 | left atrium | II | II | No | No | Positive | NA |
| 45 | ARMS | M | 4 | 4 | right hand | I | Ⅲ | Yes | Yes | Positive | NA |
| 46 | ARMS | M | 69 | 4 | left temporal | II | II | No | No | Positive | NA |
| 47 | ARMS | M | 28 | 15 | left calf | Ⅲ | II | Yes | No | Positive | NA |
| 48 | ARMS | M | 7 | 1.1 | right hand | Ⅲ | II | No | Yes | Negative | NA |
| 49 | ERMS | F | 13 | 7.4 | right lung | II | I | No | No | Negative | NA |
| 50 | ERMS | M | 29 | 10 | pelvic cavity | II | I | No | No | Negative | NA |
| 51 | ERMS | M | 4 | 3 | lips | II | I | No | No | Negative | NA |
| 52 | ERMS | F | 0.5 | 4 | vaginal | I | I | No | No | Negative | NA |
| 53 | ERMS | M | 29 | 2.5 | prostate | Ⅲ | II | No | No | Negative | NA |
| 54 | PRMS | M | 56 | 12 | left thigh | I | II | No | No | Negative | NA |
| 55 | ARMS | F | 65 | 6 | lung | II | II | No | No | Positive | NA |
| 56 | ARMS | M | 69 | 4 | right temporal | NA | NA | NA | NA | PQ | NA |
| 57 | ARMS | M | 0.58 | 2.2 | left nasal vestibule | NA | NA | NA | NA | PQ | NA |
| 58 | ARMS | F | 11 | 4 | right cheek | NA | NA | NA | NA | PQ | NA |
| 59 | ARMS | M | 25 | 5 | right elbow | NA | NA | NA | NA | PQ | NA |
| 60 | ERMS | F | 8 | 2 | right pharynx | I | Ⅲ | Yes | No | Negative | NA |
| 61 | ARMS | M | 28 | 6 | right jaw | Ⅲ | II | Yes | Yes | Positive | NA |
| 62 | ARMS | M | 18 | 5 | left forearm | Ⅲ | II | Yes | Yes | Negative | NA |
| 63 | ERMS | M | 40 | 5 | left nasal cavity | I | Ⅲ | Yes | No | Negative | NA |
| 64 | ERMS | M | 28 | 0.5 | jaw | I | Ⅲ | No | No | Negative | NA |
| 65 | ERMS | M | 20 | 6 | right face | II | II | No | No | Negative | NA |
| 66 | ARMS | M | 16 | 1 | left neck | Ⅳ | Ⅲ | No | Yes | Positive | NA |
| 67 | PRMS | F | 66 | 6 | right forearm | I | Ⅲ | No | No | Negative | NA |
| 68 | ARMS | M | 29 | 13.5 | thenar | Ⅲ | Ⅲ | No | No | Positive | Positive |
| 69 | ARMS | M | 7 | 5 | left testicle | I | II | No | No | Positive | Positive |
| 70 | ARMS | F | 24 | 3.5 | right nasal maxillary sinus | II | II | No | Yes | Positive | Positive |
| 71 | ARMS | M | 20 | 4 | left forearm | I | II | No | No | Positive | Positive |
| 72 | ARMS | F | 30 | 8 | left maxillary sinus | Ⅲ | II | No | No | Positive | Positive |
| 73 | ARMS | M | 35 | 2.5 | right testicle | I | II | No | Yes | Positive | Positive |
| 74 | ERMS | M | 33 | 0.1 | right infratemporal fossa | I | I | No | No | Negative | Negative |
| 75 | ARMS | F | 50 | 3 | right sinus | II | II | Yes | No | Positive | Positive |
| 76 | ERMS | F | 57 | 0.8 | abdominal cavity | I | II | No | No | Negative | Negative |
| 77 | ARMS | M | 5 | 3.5 | left thigh | II | II | No | No | Positive | Positive |
| 78 | PRMS | M | 69 | 1.2 | throat | I | I | No | No | Negative | NA |
| 79 | ERMS | M | 89 | 0.1 | left inguinal lymph node | I | II | No | No | Negative | NA |
| 80 | ERMS | F | 5 | 4.6 | left shoulder | I | II | No | No | Negative | NA |
| 81 | ERMS | F | 8 | 20 | left shoulder deltoid | Ⅲ | II | No | No | Negative | NA |
| 82 | PRMS | F | 74 | 11 | pelvic cavity | Ⅲ | II | No | No | Negative | NA |
| 83 | SRMS | M | 62 | 5 | neck | Ⅲ | II | No | No | Negative | NA |
| 84 | ERMS | M | 2 | 3.5 | right testicle | I | II | No | No | Negative | NA |
| 85 | ARMS | M | 5 | 1.5 | left thigh | I | Ⅲ | No | No | Positive | Positive |
| 86 | ARMS | M | 4 | 0.3 | left supraclavicular lymph node | I | II | No | No | Positive | NA |
| 87 | ARMS | M | 26 | 13.7 | left testicle | Ⅲ | II | No | No | Positive | NA |
| 88 | PRMS | M | 43 | 9 | left hip | II | II | No | No | Negative | NA |
| 89 | PRMS | F | 72 | 9.3 | left forearm | II | Ⅲ | No | No | Negative | NA |
| 90 | PRMS | F | 41 | 11 | right calf | Ⅲ | II | No | No | Negative | NA |
| 91 | PRMS | M | 84 | 5 | the back | I | II | No | No | Negative | NA |
| 92 | ARMS | F | 65 | 0.3 | left nasal cavity | Ⅲ | II | Yes | No | Positive | Positive |
| 93 | ERMS | M | 27 | 4 | infratemporal fossa | I | I | No | No | Negative | Negative |
| 94 | ERMS | F | 17 | 5 | right parotid gland | Ⅲ | I | No | No | Negative | Negative |
| 95 | PRMS | F | 61 | 13 | waist | Ⅲ | Ⅲ | No | No | Negative | NA |
| 96 | ARMS | F | 7 | 3.8 | spinal canal | I | II | No | No | Positive | NA |
| 97 | PRMS | M | 63 | 11 | left thigh | Ⅲ | Ⅲ | No | No | Negative | NA |
| 98 | pPNET | F | 59 | 10 | right shoulder | NA | NA | NA | NA | Positive | NA |
| 99 | pPNET | M | 17 | 12 | calf | NA | NA | NA | NA | Positive | NA |
| 100 | pPNET | M | 21 | 2.5 | right groin | NA | NA | NA | NA | Positive | NA |
| 101 | pPNET | F | 17 | 3.7 | right forearm | NA | NA | NA | NA | PQ | NA |
| 102 | pPNET | F | 35 | 10 | right forearm | NA | NA | NA | NA | Negative | NA |
| 103 | pPNET | F | 19 | 4 | left upper arm | NA | NA | NA | NA | Negative | NA |
| 104 | pPNET | F | 22 | 9 | right retroperitoneum | NA | NA | NA | NA | Positive | NA |
| 105 | pPNET | M | 10 | 10 | pelvic cavity | NA | NA | NA | NA | Positive | NA |
| 106 | pPNET | M | 33 | 7 | right thigh | NA | NA | NA | NA | Positive | NA |
| 107 | pPNET | F | 17 | 3.7 | right forearm | NA | NA | NA | NA | Positive | NA |
| 108 | pPNET | F | 28 | 2 | left neck | NA | NA | NA | NA | Positive | NA |
| 109 | pPNET | M | 23 | 5 | manubrium | NA | NA | NA | NA | Negative | NA |
| 110 | pPNET | F | 46 | 3 | right brachial plexus | NA | NA | NA | NA | Negative | NA |
| 111 | pPNET | F | 14 | 12 | left shoulder blade | NA | NA | NA | NA | Positive | NA |
| 112 | pPNET | F | 13 | 7 | right chest back | NA | NA | NA | NA | Negative | NA |
| 113 | pPNET | M | 20 | 9 | left rib | NA | NA | NA | NA | Negative | NA |
| 114 | pPNET | M | 27 | 6 | left thigh | NA | NA | NA | NA | Negative | NA |
| 115 | pPNET | M | 24 | 16 | iliac | NA | NA | NA | NA | Positive | NA |
| 116 | pPNET | F | 26 | 10 | right thigh | NA | NA | NA | NA | Negative | NA |
| 117 | pPNET | M | 13 | 15 | left thigh | NA | NA | NA | NA | Negative | NA |
| 118 | pPNET | F | 25 | 20 | left elbow | NA | NA | NA | NA | Negative | NA |
| 119 | pPNET | F | 13 | 2 | left neck | NA | NA | NA | NA | Positive | NA |
| 120 | pPNET | M | 13 | 6 | right forearm | NA | NA | NA | NA | Positive | NA |
| 121 | pPNET | M | 41 | 2.5 | left wrist | NA | NA | NA | NA | Positive | NA |
| 122 | pPNET | M | 21 | 8 | right testicle | NA | NA | NA | NA | Positive | NA |
| 123 | pPNET | M | 19 | 3.4 | right ankle | NA | NA | NA | NA | Positive | NA |
| 124 | pPNET | F | 25 | 20 | retroperitoneum | NA | NA | NA | NA | Positive | NA |
| 125 | pPNET | F | 59 | 10 | right shoulder | NA | NA | NA | NA | PQ | NA |
| 126 | pPNET | M | 17 | 12 | calf | NA | NA | NA | NA | PQ | NA |
| 127 | pPNET | F | 36 | 2.5 | instep | NA | NA | NA | NA | PQ | NA |
| 128 | pPNET | M | 15 | 4 | retroperitoneum | NA | NA | NA | NA | PQ | NA |
| 129 | pPNET | F | 16 | 12 | left hip | NA | NA | NA | NA | PQ | NA |
| 130 | pPNET | M | 27 | 6 | right elbow | NA | NA | NA | NA | PQ | NA |
| 131 | pPNET | F | 17 | 3.7 | right forearm | NA | NA | NA | NA | PQ | NA |
| 132 | pPNET | M | 21 | 2.5 | right inguinal testis | NA | NA | NA | NA | PQ | NA |
| 133 | pPNET | M | 24 | 13 | iliac | NA | NA | NA | NA | PQ | NA |
| 134 | pPNET | F | 37 | 1 | right paravertebral | NA | NA | NA | NA | PQ | NA |
| 135 | pPNET | M | 26 | 14 | left thigh | NA | NA | NA | NA | PQ | NA |
| 136 | pPNET | M | 23 | 11 | head and neck | NA | NA | NA | NA | PQ | NA |
| 137 | pPNET | M | 28 |  | left ethmoid sinus | NA | NA | NA | NA | PQ | NA |
| 138 | pPNET | F | 13 | 4 | left neck | NA | NA | NA | NA | PQ | NA |
| 139 | pPNET | M | 52 | 6 | right shin anterior | NA | NA | NA | NA | Positive | NA |
| 140 | BSS | F | 32 | 8 | left groin | NA | NA | NA | NA | Positive | NA |
| 141 | BSS | F | 38 | 5.5 | left forearm | NA | NA | NA | NA | Positive | NA |
| 142 | BSS | F | 15 | 4 | right neck | NA | NA | NA | NA | Positive | NA |
| 143 | MFSS | M | 14 | 6 | left elbow fossa | NA | NA | NA | NA | Positive | NA |
| 144 | BSS | F | 43 | 13 | left hip | NA | NA | NA | NA | Positive | NA |
| 145 | BSS | F | 40 | 2.8 | right thigh | NA | NA | NA | NA | Positive | NA |
| 146 | BSS | F | 22 | 3 | left heel | NA | NA | NA | NA | Positive | NA |
| 147 | BSS | M | 19 | 8.5 | left calf ankle | NA | NA | NA | NA | Positive | NA |
| 148 | BSS | F | 10 | 5 | behind the right elbow | NA | NA | NA | NA | Positive | NA |
| 149 | BSS | M | 36 | 5 | left thigh | NA | NA | NA | NA | Positive | NA |
| 150 | MFSS | F | 55 | 5 | left iliac wing | NA | NA | NA | NA | Positive | NA |
| 151 | BSS | M | 36 | 11 | right elbow | NA | NA | NA | NA | Positive | NA |
| 152 | BSS | F | 37 | 6 | upper right tibia | NA | NA | NA | NA | Positive | NA |
| 153 | BSS | M | 33 | 4 | left lung | NA | NA | NA | NA | Positive | NA |
| 154 | BSS | F | 25 | 3 | left knee joint | NA | NA | NA | NA | Positive | NA |
| 155 | BSS | M | 26 | 1.4 | left popliteal fossa | NA | NA | NA | NA | PQ | NA |
| 156 | BSS | F | 37 | 5.5 | left neck | NA | NA | NA | NA | Positive | NA |
| 157 | BSS | F | 28 | 7 | right parotid gland | NA | NA | NA | NA | Positive | NA |
| 158 | BSS | M | 23 | 10.5 | right upper abdomen | NA | NA | NA | NA | Positive | NA |
| 159 | BSS | M | 61 | 3 | L5 vertebral body | NA | NA | NA | NA | Positive | NA |
| 160 | MFSS | F | 29 | 16 | lower back | NA | NA | NA | NA | Positive | NA |
| 161 | BSS | F | 70 | 15 | right calf | NA | NA | NA | NA | Positive | NA |
| 162 | MFSS | M | 37 | 2 | right knee joint | NA | NA | NA | NA | Positive | NA |
| 163 | MFSS | M | 70 | 7 | right calf | NA | NA | NA | NA | Positive | NA |
| 164 | MFSS | M | 40 | 22 | right groin | NA | NA | NA | NA | Positive | NA |
| 165 | BSS | F | 15 | 6.5 | left second toe | NA | NA | NA | NA | Positive | NA |
| 166 | BSS | F | 38 | 5 | left scapula | NA | NA | NA | NA | Positive | NA |
| 167 | BSS | M | 24 | 5 | right thumb | NA | NA | NA | NA | Positive | NA |
| 168 | BSS | M | 74 | 5 | left hip | NA | NA | NA | NA | PQ | NA |
| 169 | PDFF | F | 41 | 3.2 | right anterior lobe of liver | NA | NA | NA | NA | Positive | NA |
| 170 | MFSS | M | 35 | 5 | right distal femur | NA | NA | NA | NA | Positive | NA |
| 171 | MFSS | F | 19 | 7.5 | left lesser trochanter | NA | NA | NA | NA | Positive | NA |
| 172 | MFSS | M | 47 | 5 | left tibia | NA | NA | NA | NA | Positive | NA |
| 173 | BSS | M | 70 | 11.5 | left hip | NA | NA | NA | NA | Positive | NA |
| 174 | BSS | M | 39 | 6 | left hand and forearm | NA | NA | NA | NA | Positive | NA |
| 175 | BSS | F | 21 | 7.5 | right thigh | NA | NA | NA | NA | Positive | NA |
| 176 | MFSS | M | 32 | 3 | lower right posterior region | NA | NA | NA | NA | Positive | NA |
| 177 | BSS | M | 55 | 5.2 | right foot | NA | NA | NA | NA | Positive | NA |
| 178 | MFSS | M | 40 | 1 | right elbow | NA | NA | NA | NA | PQ | NA |
| 179 | PDFF | M | 13 | 7.5 | left elbow | NA | NA | NA | NA | PQ | NA |
| 180 | MFSS | M | 14 | 5 | left elbow fossa | NA | NA | NA | NA | Positive | NA |
| 181 | PDFF | M | 17 | 6 | left elbow fossa | NA | NA | NA | NA | Positive | NA |
| 182 | PDFF | M | 3 | 1 | thoracic | NA | NA | NA | NA | Positive | NA |
| 183 | BSS | M | 36 | 8.3 | left thigh | NA | NA | NA | NA | Positive | NA |
| 184 | BSS | F | 22 | 7 | left foot | NA | NA | NA | NA | Positive | NA |
| 185 | BSS | M | 54 | 6 | left nipple | NA | NA | NA | NA | Positive | NA |
| 186 | BSS | F | 12 | 7.3 | right elbow | NA | NA | NA | NA | Positive | NA |
| 187 | PDFF | M | 13 | 10 | left elbow | NA | NA | NA | NA | Positive | NA |
| 188 | PDFF | M | 13 | 4 | left axillary lymph node metastasis | NA | NA | NA | NA | Negative | NA |
| 189 | PDFF | F | 14 | 3 | metastasis of the right elbow to the marrow cavity | NA | NA | NA | NA | Positive | NA |
| 190 | BSS | M | 14 | 15 | upper right calf | NA | NA | NA | NA | Positive | NA |
| 191 | BSS | F | 12 | 2.3 | right index finger | NA | NA | NA | NA | Negative | NA |
| 192 | BSS | M | 50 | 17 | right upper arm | NA | NA | NA | NA | Positive | NA |
| 193 | BSS | F | 24 | 4 | left instep | NA | NA | NA | NA | Positive | NA |
| 194 | BSS | M | 27 | 3 | right heel | NA | NA | NA | NA | Positive | NA |
| 195 | MFSS | F | 20 | 3.5 | right occiput | NA | NA | NA | NA | Positive | NA |
| 196 | MFSS | M | 22 | 14 | left popliteal fossa | NA | NA | NA | NA | Positive | NA |
| 197 | MFSS | M | 19 | 9 | left popliteal fossa | NA | NA | NA | NA | Positive | NA |
| 198 | BSS | F | 24 | 4 | left instep | NA | NA | NA | NA | Positive | NA |
| 199 | BSS | M | 27 | 3 | left heel | NA | NA | NA | NA | Positive | NA |
| 200 | MFSS | M | 32 | 5.5 | right knee joint | NA | NA | NA | NA | Positive | NA |
| 201 | MFSS | M | 15 | 13 | right elbow | NA | NA | NA | NA | PQ | NA |
| 202 | MFSS | F | 40 | 4 | left popliteal fossa | NA | NA | NA | NA | PQ | NA |
| 203 | MFSS | M | 52 | 5 | right kidney | NA | NA | NA | NA | Positive | NA |
| 204 | MFSS | M | 51 | 10 | left upper limb | NA | NA | NA | NA | Positive | NA |
| 205 | MFSS | M | 17 | 7 | right upper limb | NA | NA | NA | NA | Positive | NA |
| 206 | MFSS | F | 56 | 2.5 | right lower limb | NA | NA | NA | NA | Negative | NA |
| 207 | MFSS | M | 56 | 11 | lung | NA | NA | NA | NA | Positive | NA |
| 208 | MFSS | F | 48 | 10 | right lower lobe | NA | NA | NA | NA | Positive | NA |
| 209 | MFSS | F | 46 | 4.5 | neck | NA | NA | NA | NA | Positive | Positive |
| 210 | MFSS | M | 70 | 1 | left upper arm | NA | NA | NA | NA | Positive | Positive |
| 211 | ASPS | F | 36 | 3.5 | left thigh | NA | NA | NA | NA | Positive | Positive |
| 212 | ASPS | M | 34 | 6.5 | right elbow | NA | NA | NA | NA | Positive | NA |
| 213 | ASPS | F | 26 | 11 | left thigh | NA | NA | NA | NA | Positive | NA |
| 214 | ASPS | M | 36 | 1.5 | right eye socket | NA | NA | NA | NA | Positive | NA |
| 215 | ASPS | F | 3 |  | right eye socket | NA | NA | NA | NA | PQ | NA |
| 216 | ASPS | F | 10 | 7 | right eye socket | NA | NA | NA | NA | PQ | NA |
| 217 | ASPS | F | 11 | 5 | right thigh | NA | NA | NA | NA | PQ | NA |
| 218 | ASPS | F | 58 | 2 | right chest wall | NA | NA | NA | NA | Positive | NA |
| 219 | ASPS | M | 20 | 4 | right breast | NA | NA | NA | NA | PQ | NA |
| 220 | ASPS | F | 18 | 7.5 | left thigh | NA | NA | NA | NA | Positive | NA |
| 221 | ASPS | F | 32 | 6 | renal hilum area | NA | NA | NA | NA | Positive | NA |
| 222 | ASPS | F | 32 | 5 | left upper arm | NA | NA | NA | NA | Positive | NA |
| 223 | ASPS | F | 17 | 5.4 | brain | NA | NA | NA | NA | Positive | NA |
| 224 | ASPS | F | 26 | 10 | left thigh | NA | NA | NA | NA | Positive | NA |
| 225 | MLPS | F | 51 | 5 | left thigh | NA | NA | NA | NA | Positive | NA |
| 226 | MLPS | F | 40 | 21 | retroperitoneum | NA | NA | NA | NA | Negative | NA |
| 227 | MLPS | F | 42 | 3.8 | spinal canal | NA | NA | NA | NA | Positive | NA |
| 228 | MLPS | F | 41 | 12.7 | left thigh | NA | NA | NA | NA | Positive | NA |
| 229 | MLPS | M | 74 | 20 | left thigh | NA | NA | NA | NA | Positive | NA |
| 230 | DFSP | F | 8 | 6.5 | abdominal wall | NA | NA | NA | NA | Positive | NA |
| 231 | DFSP | M | 74 | 4.5 | waist | NA | NA | NA | NA | Negative | NA |
| 232 | DFSP | M | 6 | 5.5 | the back | NA | NA | NA | NA | Positive | NA |
| 233 | DFSP | M | 28 | 5 | left arm | NA | NA | NA | NA | Positive | NA |
| 234 | DFSP | F | 23 | 4.5 | left shoulder | NA | NA | NA | NA | Positive | NA |
| 235 | DFSP | F | 48 | 6 | groin | NA | NA | NA | NA | Positive | NA |
| 236 | DFSP | F | 21 | 5.3 | left shoulder | NA | NA | NA | NA | Negative | NA |
| 237 | DFSP | F | 29 | 8 | left breast | NA | NA | NA | NA | Positive | NA |
| 238 | DFSP | M | 47 | 10 | right leg | NA | NA | NA | NA | Positive | NA |
| 239 | DFSP | M | 32 | 7 | abdominal wall | NA | NA | NA | NA | Positive | NA |
| 240 | DFSP | M | 38 | 9 | the back | NA | NA | NA | NA | Negative | NA |
| 241 | DFSP | M | 43 | 11 | right lower abdomen | NA | NA | NA | NA | Negative | NA |
| 242 | AFST | M | 13 | 6.2 | left thigh | NA | NA | NA | NA | Positive | Positive |

**Note:** ARMS: Alveolar rhabdomyosarcoma; ERMS: Embryonal rhabdomyosarcoma; Pleomorphic rhabdomyosarcoma; SRMS: Sclerosing rhabdomyosarcoma;

pPNET: peripheral Primitive neuroectodermal tumor; BSS: Biphasic synovial sarcoma; MFSS: Monophasic fibrous synovial sarcoma; PDSS: Poorly differentiated synovial sarcoma;

ASPS: Alveolar soft tissue sarcoma; MLPS : Myxoid liposarcoma; DFSP: Dermatofibrosarcoma protuberans; AFST: Soft-tissue angiofibroma; PQ: Poor quality of RNA. NA: Not available
